# Progranulin regulates neuronal outgrowth independent of Sortilin

**DOI:** 10.1186/1750-1326-7-33

**Published:** 2012-07-10

**Authors:** Jennifer Gass, Wing C Lee, Casey Cook, Nicole Finch, Caroline Stetler, Karen Jansen-West, Jada Lewis, Christopher D Link, Rosa Rademakers, Anders Nykjær, Leonard Petrucelli

**Affiliations:** 1 Department of Neuroscience, Mayo Clinic College of Medicine, 4500 San Pablo Road, Jacksonville, Florida, 32224, USA; 2 Department of Neuroscience and Center for Translational Research in Neurodegenerative Disease, University of Florida, Gainesville, FL, 32610, USA; 3 Integrative Physiology Institute for Behavioral Genetics, University of Colorado, UBC447, Boulder, CO, 80309, USA; 4 The Lundbeck Foundation Research Center MIND, Department of Medical Biochemistry, Aarhus University, Aarhus, Denmark

**Keywords:** Progranulin, Sortilin, Neuronal outgrowth, Frontotemporal lobar degeneration, Neurotrophic factor

## Abstract

**Background:**

Progranulin (PGRN), a widely secreted growth factor, is involved in multiple biological functions, and mutations located within the PGRN gene (*GRN*) are a major cause of frontotemporal lobar degeneration with TDP-43-positive inclusions (FLTD-TDP). In light of recent reports suggesting PGRN functions as a protective neurotrophic factor and that sortilin (SORT1) is a neuronal receptor for PGRN, we used a Sort1-deficient (*Sort1*^*−/−*^) murine primary hippocampal neuron model to investigate whether PGRN’s neurotrophic effects are dependent on SORT1. We sought to elucidate this relationship to determine what role SORT1, as a regulator of PGRN levels, plays in modulating PGRN’s neurotrophic effects.

**Results:**

As the first group to evaluate the effect of PGRN loss in *Grn* knockout primary neuronal cultures, we show neurite outgrowth and branching are significantly decreased in *Grn*^*−/−*^ neurons compared to wild-type (WT) neurons. More importantly, we also demonstrate that PGRN overexpression can rescue this phenotype. However, the recovery in outgrowth is not observed following treatment with recombinant PGRN harboring missense mutations p.C139R, p.P248L or p.R432C, indicating that these mutations adversely affect the neurotrophic properties of PGRN. In addition, we also present evidence that cleavage of full-length PGRN into granulin peptides is required for increased neuronal outgrowth, suggesting that the neurotrophic functions of PGRN are contained within certain granulins. To further characterize the mechanism by which PGRN impacts neuronal morphology, we assessed the involvement of SORT1. We demonstrate that PGRN induced-outgrowth occurs in the absence of SORT1 in *Sort1*^*−/−*^ cultures.

**Conclusion:**

We demonstrate that loss of PGRN impairs proper neurite outgrowth and branching, and that exogenous PGRN alleviates this impairment. Furthermore, we determined that exogenous PGRN induces outgrowth independent of SORT1, suggesting another receptor(s) is involved in PGRN induced neuronal outgrowth.

## Background

Frontotemporal lobar degeneration (FTLD) is the second-most common presenile dementia after Alzheimer’s disease (AD) [1]. FTLD is neuropathologically characterized by predominant atrophy of the frontal and temporal lobes and the presence of proteinaceous inclusions in neurons and glial cells [2]. FTLD cases are divided into two main pathological subgroups at autopsy: those with tau-positive inclusions (FTLD-tau), and those with TDP-43-positive inclusions (FTLD-TDP) [3].

In 2006, mutations located within *GRN*, were described as a major cause of FTLD-TDP [4,5]. Pathogenic mutations in *GRN* mainly include frameshift, splice site and nonsense mutations that produce a premature termination of the coding sequence and consequently lead to degradation of mutant mRNA via nonsense-mediated decay [4-6]. As a result, pathogenic mutations are thought to cause disease by *GRN* haploinsufficiency. With the exception of the pathogenic missense mutation p.A9D, which prevents PGRN secretion, it is not known whether missense mutations in *GRN* influence PGRN’s neurotrophic properties [6,7].

PGRN is a widely expressed secreted growth factor involved in development, wound repair and inflammation [8,9]. Recent studies suggest that PGRN functions as a protective neurotrophic factor; however, its role in the CNS is not well understood [10-13]. The mechanisms underlying its effects have not been fully determined, nor is it known how missense mutations in *GRN* influence its neurotrophic properties.

The protein contains seven-and-a-half tandem repeats of a 12-cysteine granulin motif separated by interlinked spacer regions that are termed granulins [14]. In the periphery, full-length PGRN is proteolytically cleaved into mature granulins via proteases [15,16]. Secretory leukocyte protease inhibitor (SLPI), a serine protease inhibitor, prevents PGRN cleavage by binding to PGRN cleavage sites or to elastase and inhibiting its cleavage activity [16].

Recently, two PGRN receptors have been identified: sortilin 1 (SORT1) and tumor necorosis factor receptor (TNFR) [17,18]. The neuronal PGRN receptor, SORT1, has been shown to regulate the extracellular levels of PGRN [17,19]. SORT1, a vacuolar protein sorting 10 protein domain receptor, is also involved in endocytosis and transport of proteins to the trans-golgi network and endosomes/lysosomes [20]. The C-terminal portion of PGRN binds to SORT1 causing immediate endocytosis of PGRN to the lysosome. Due to its interactions with SORT1, we sought to determine whether PGRN utilizes this receptor to modulate neuronal morphology. With regard to the loss of PGRN leading to neurodegeneration, we investigated the physiological impact/consequences of loss of PGRN in primary hippocampal neuronal cultures.

## Results

### Loss of PGRN alters neuronal morphology

**Figure 1:**
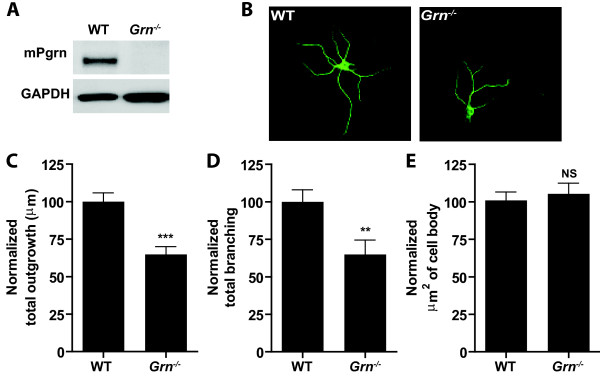
**Loss of Pgrn decreases neurite outgrowth and branching in primary neurons.** (**A**) Western blot analysis reveals endogenous levels of Pgrn in WT and *Grn*^*−/−*^ primary hippocampal cultures. (**B**) WT and *Grn*^*−/−*^ hippocampal neurons immunostained for MAP2. (**C-E**). Neurite outgrowth and branching of MAP2-immunopositive neurons was assessed using MetaMorph version 7.1 software. Note that neurite outgrowth (**C**) and branching (**D**), but not cell body size (**E**), are decreased in *Grn*^*−/−*^ hippocampal neurons compared to WT hippocampal neurons. All data is presented as the mean ± SEM. **p < 0.01; ***p < 0.001.

*GRN* haploinsufficiency due to mutations in *GRN* cause FTLD-TDP [2]. To evaluate the impact of PGRN deficiency on neuron morphology, primary hippocampal cultures from WT and *Grn*^*−/−*^ mice were prepared. Western blot analysis confirmed the complete loss of PGRN in hippocampal cultures from *Grn*^*−/−*^ mice (Figure 1A). In order to assess neuronal outgrowth, neurons were fixed and immunostained using neuron-specific anti-MAP2 following 10 days in vitro (DIV10). Using confocal microscopy, we captured and analyzed images using MetaMorph 7.1 software (Figure 1B), which quantitatively measures total skeletonized outgrowth of individual neurons in μms and total branching junctions of dendrites. As quantified in Figure 1C-E, *Grn*^*−/−*^ neurons displayed significantly reduced neurite outgrowth (p < 0.0001) and branching (p < 0.005) compared to WT neurons, while cell body size remained the same. In all experiments, at least 100 neurons were analyzed and repeated in three independent experiments.

### Exogenous PGRN rescues neurite outgrowth and branching in *Grn*^*−/−*^ neurons

Having established that a loss of endogenous PGRN causes major deficits in neurite outgrowth and branching of hippocampal neurons, we then examined whether this phenotype can be rescued by returning PGRN to cultures. To achieve PGRN overexpression in our cultures, we packaged PGRN into a rAAV1 vector (rAAV1-PGRN). Dosing titers are expressed genomes/mL.

**Figure 2:**
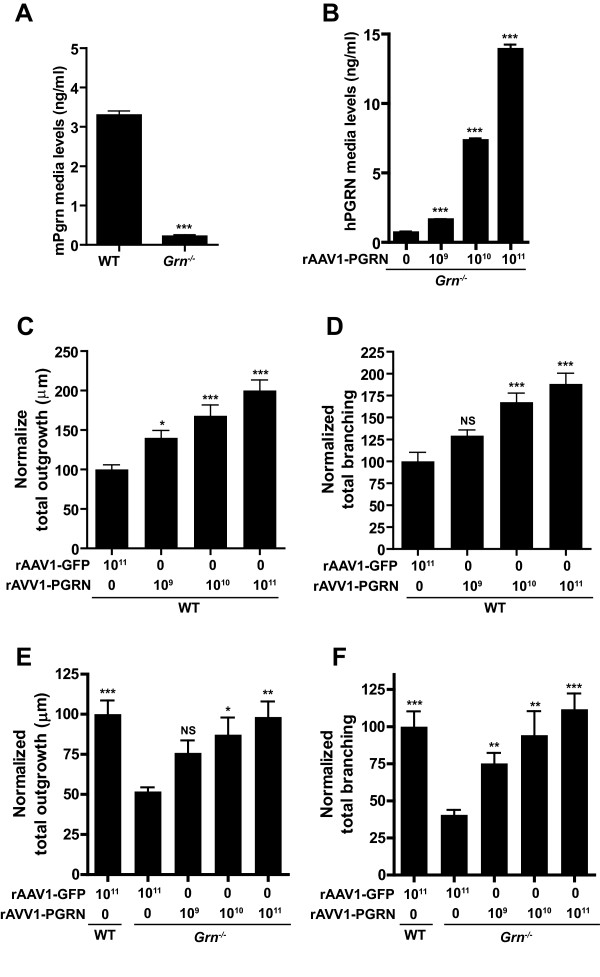
**Primary hippocampal cultures transduced with rAAV1-PGRN promotes neurite outgrowth and branching.** (**A**) Mouse Pgrn ELISA of endogenous Pgrn levels in hippocampal primary neuronal cultures. (**B**) Human PGRN ELISA shows increasing levels of hPGRN generated in the media of treated primary cultures. (**C,D**) Bar graph quantification of total outgrowth (**C**) and branching (**D**)of primary hippocampal neurons transduced with increasing amounts of rAAV1-PGRN in WT cultures normalized to WT rAAV1-GFP treated cultures. (**E,F**) Bar graph quantification of normalized total outgrowth (**E**) and branching (**F**)in *Grn*^*−/−*^ hippocampal neurons transduced with rAAV1-PGRN normalized to WT rAAV1-GFP treated cultures. Note that rAAV1-GFP was supplemented in rAAV1-PGRN non-treated cultures. Data is presented as the mean ± SEM. *p < 0.05; ***p < 0.001.

To determine if we could rescue the *Grn*^*−/−*^ phenotype, we transduced primary hippocampal neurons with increasing doses of rAAV1-PGRN for 6 days to allow for synthesis of PGRN. Under normal conditions, our WT primary culture’s Pgrn levels were around 4 ng/mL (Figure 2A). In testing for the production and release of PGRN into the media, human specific ELISA revealed a dose-dependent increase of PGRN secreted into the medium (Figure 2B). Neuronal morphology was then assessed in *Grn*^*−/−*^ and WT neurons treated with the same increasing titers of rAAV1-PGRN. Application of rAAV1-PGRN resulted in statistically significant increased outgrowth and branching in both *Grn*^*−/−*^ and WT cultures (Figure 2C-F). rAAV1-GFP transduction in cultures did not promote outgrowth or branching, suggesting that the production of PGRN induces outgrowth and plays a role in neuronal morphology. All conditions were normalized to WT rAAV1-GFP treated cells revealing that the phenotype was rescued at a rAAV1-PGRN titer of 10^11^ genomes/mL or 14 ng/mL within the media.

### rPGRN promotes neuronal outgrowth and branching in *Grn*^*−/−*^ and WT cultures

**Figure 3:**
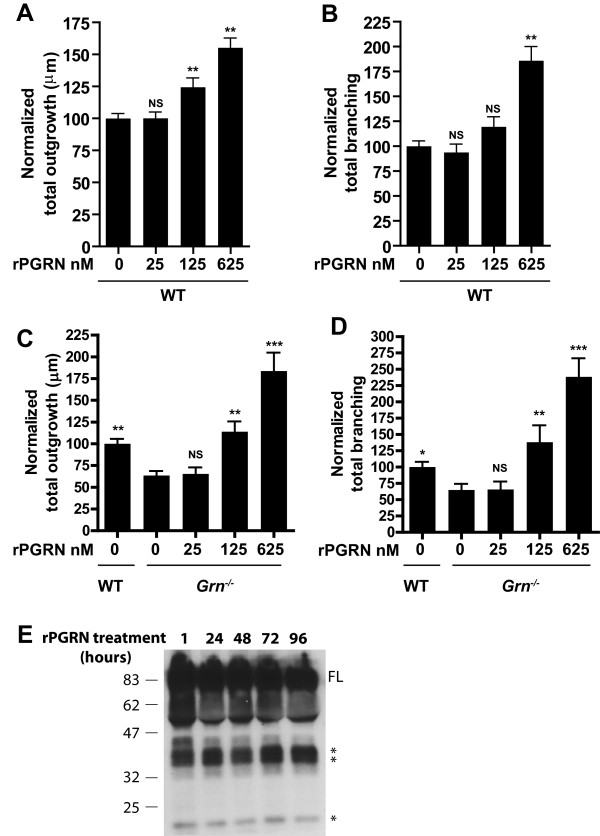
**rPGRN promotes neurite outgrowth and branching in primary neurons.** (**A**) (**A,B**) Bar graph quantification of total neurite outgrowth (**A**) and branching (**B**) of WT hippocampal neurons exposed to rPGRN increasing 25, 125, 625 nM doses of His-rPGRN (nM doses correspond to 2,10, 50 ug/mL concentrations respectively). All doses normalized to WT non-treated cultures. (**C,D**) Bar graph quantification of normalized total neurite outgrowth (**C**) and branching (**D**) in of *Grn*^*−/−*^ hippocampal neurons exposed to rPGRN. *Grn*^*−/−*^ cultures were normalized to WT non-treated cultures. (**E**) Western blot analysis reveals PGRN cleavage in *Grn*^*−/−*^ hippocampal cultures treated with 125 nM rPGRN-His at increasing time points. Note cleavage of PGRN after only one hour. Data is presented as the mean ± SEM. **p < 0.01.

Since determining that exogenous overexpression of rAAV1-PGRN is able to rescue the *Grn*^*−/−*^ neuronal phenotype, we purified recombinant human PGRN (rPGRN) from media of a stable HEK 293 cell line overexpressing PGRN. The rPGRN was then applied to WT and *Grn*^*−/−*^ cultures in increasing doses. The results from this experiment show a dose-dependent, significant increase in outgrowth and branching in *Grn*^*−/−*^ neurons as well as WT neurons (Figure 3A-D). These results also reveal that 125 nM (10 ug/mL) rPGRN is needed to rescue the *Grn*^*−/−*^ phenotype and return outgrowth and branching to normal conditions.

### Cleavage of PGRN promotes neuronal outgrowth

**Figure 4:**
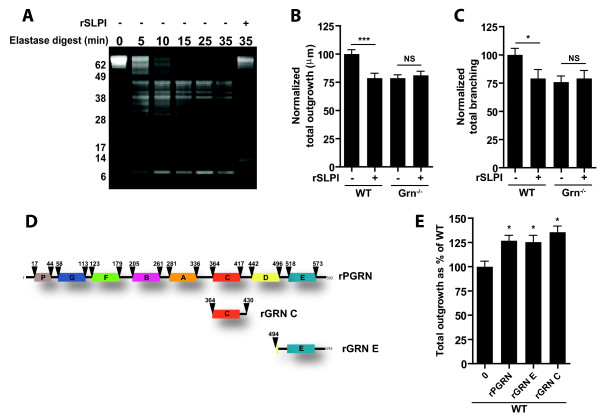
**PGRN processing promotes neuronal outgrowth and branching.** (**A**) Time course analysis of rPGRN subjected to elastase digest. PGRN cleavage products visualized by SDS-PAGE, post-stain with sypro ruby solution (Sigma). Note that the co-treatment of recombinant SLPI blocks elastase-mediated rPGRN cleavage. (**B,C**) Bar graphs depict quantification of total neurite outgrowth (**B**) and branching (**C**) of WT and *Grn*^*−/−*^ neurons treated with or without rSLPI. Note that in these experiments no rPGRN was added, therefore rSLPI blocks endogenous Pgrn. (**D**) Schematic representation of recombinant proteins analyzed in primary hippocampal neurons. (**E**) WT hippocampal cultures treated with recombinant granulin E (rGRN E) and granulin C (rGRN C) promote outgrowth similar to full length PGRN Data is presented as the mean ± SEM. *p < 0.05; ***p < 0.001.

PGRN is secreted as a full-length 88 kDa protein, which is cleaved into mature granulins. In fact, Western blot analysis of rPGRN treated cultures shows rPGRN cleavage within cultures (Figure 3E). Note that in the media, rPGRN becomes cleaved into granulin fragments within 1 hour. To determine whether PGRN or granulins are responsible for inducing neurite outgrowth, WT cultures were treated with recombinant SLPI (rSLPI), a serine protease inhibitor that prevents PGRN cleavage [16]. Utilizing an elastase digest assay, we first confirmed that rSLPI prevents processing of rPGRN into GRNs (Figure 4A). Upon incubation of rPGRN with elastase, PGRN cleavage occurred within 5 minutes; however, when rSLPI was added, the cleavage of PGRN by elastase was inhibited.

We sought to examine rSLPI’s effects on endogenous mouse Pgrn levels, which had yet to be determined. After treating WT cultures expressing endogenous Pgrn with rSLPI, we observed reduced outgrowth (Figure 4B) and branching (Figure 4C) similar to untreated *Grn*^*−/−*^ cultures. When rSLPI was added to *Grn*^*−/−*^ cultures there was no change in outgrowth or branching, illustrating that rSLPI is targeting PGRN and that PGRN cleavage is required to induce outgrowth. While treatment with rPGRN alone increases outgrowth, co-treatment of rSLPI and rPGRN in WT neurons does not increase outgrowth (Additional file 1: Figure S1).

These findings next led us to investigate the neurotrophic activity of specific granulins. Here, neurons were treated with recombinant granulin protein containing full length PGRN, and commercially available granulin E (rGRN E) or granulin C (rGRN C) (Figure 4D). Treatment with granulins E and C (125 nM/10 ug/mL) promoted neuronal outgrowth very similar to PGRN treatment (125 nM/10 ug/mL) suggesting these granulin species harbor neurotrophic properties (Figure 4E).

### C-terminal tag inhibits SORT1 uptake and PGRN endocytosis

**Figure 5:**
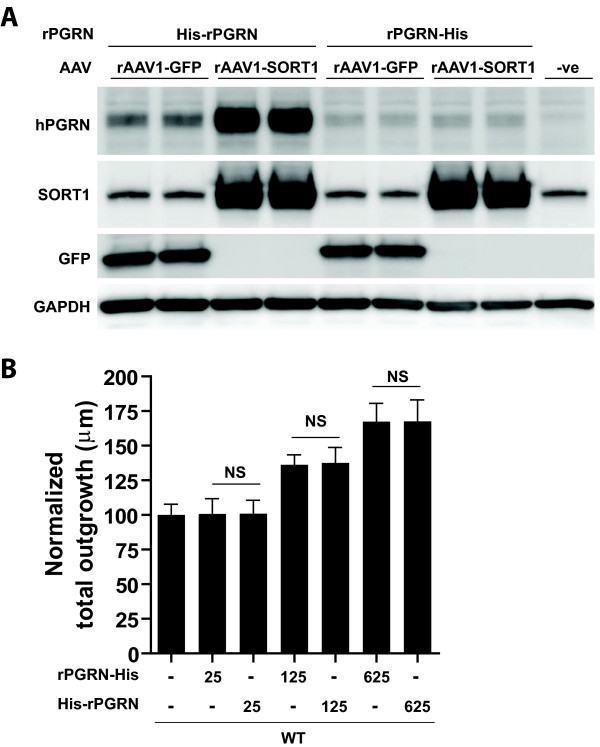
**SORT1 triggers endocytosis of rPGRN.** (**A**) Western blot analysis of Neuro2A cells transduced with rAAV-GFP or rAAV-SORT1 in the presense of His-rPGRN or rPGRN-His. Note uptake of His-rPGRN into cell lysates and the absence of rPGRN-His uptake. (**B**) Bar graph quantification of WT primary neuronal cultures treated with increasing 25, 125, 625 nM doses of His-rPGRN or rPGRN-His depict equal amounts of outgrowth with between treatments (nM doses correspond to 2, 10, 50 ug/mL concentrations respectively). Data is presented as the mean ± SEM.

SORT1 was recently identified as a neuronal receptor for PGRN; however, its role in PGRN function is not well understood. We decided to test recombinant PGRN (rPGRN) on N2A cells transduced to overexpress rAAV1-SORT1 or rAAV1-GFP (Figure 5A). Human rPGRN was generated from an HEK293 stable cell line containing a His-tag located at either the N-terminal (His-rPGRN) or C-terminal (rPGRN-His) region of rPGRN. Our results indicate that overexpression of SORT1 triggers increased uptake of His-rPGRN and not rPGRN-His, suggesting the C-terminal tag inhibits PGRN’s ability to bind to SORT. Since the binding of PGRN to SORT1 was hindered by the C-terminal tag, we next compared the ability of His-rPGRN and rPGRN-His to promote outgrowth and branching in primary hippocampal cultures. We determined that both His-rPGRN and rPGRN-His were able to equally induce outgrowth (Figure 5B).

### PGRN promotes neuronal outgrowth and branching independent of Sortilin

**Figure 6:**
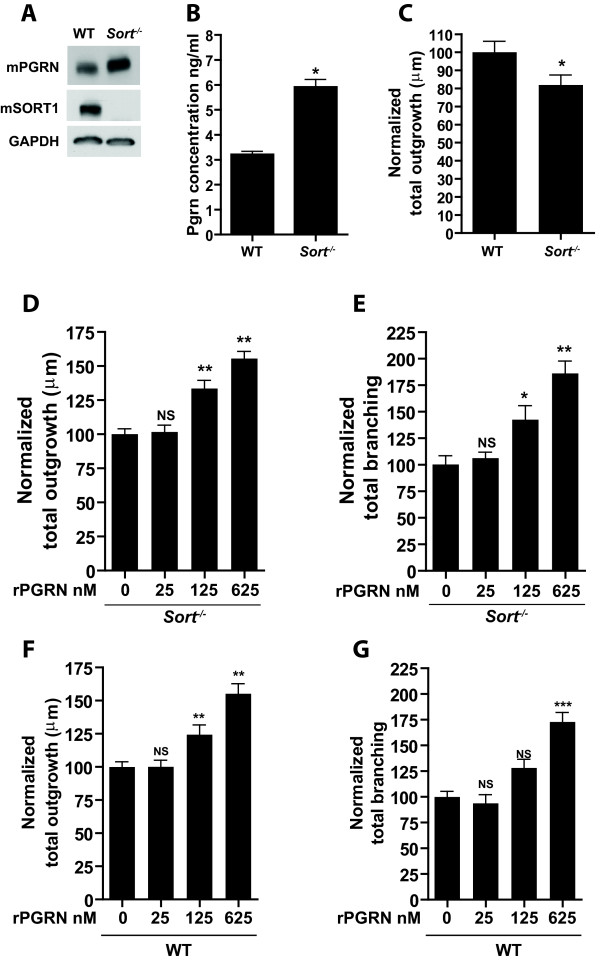
**Pgrn induces outgrowth independent of Sort1.** (**A**) Western blot analysis of Pgrn and Sort1 levels in WT and *Sort1*^*−/−*^ hippocampal cultures. (**B**) Mouse specific ELISA showing increasing levels of Pgrn in *Sort1*^*−/−*^ culture media. (**C**) Bar graph quantification of normalized total neurite outgrowth in *Sort1*^*−/−*^ and WT cultures showing significant decreases in *Sort1*^*−/−*^. (**D-G**) Normalized total outgrowth (**D,F**) and total branching (**E,G**) in *Sort1*^*−/−*^ and WT cultures with increasing amounts of His-rPGRN treatment. Data is presented as the mean ± SEM. *p < 0.05; **p < 0.01.

We previously showed that SORT1 can regulate PGRN levels, but to elucidate the role this receptor plays in neurotrophic morphology, we examined *Sort1*^*−/−*^ hippocampal cultures. Western blot analysis confirmed the complete deletion of Sort1 in hippocampal cultures (Figure 6A). Since Sort1 is involved in uptake of PGRN, we then wanted to learn whether these cultures have a marginal increase in secreted Pgrn. ELISA data from WT and *Sort1*^*−/−*^ cultures revealed increases of Pgrn in *Sort1*^*−/−*^ cultures compared to WT (Figure 6B). On the other hand, neuronal outgrowth studies revealed decreased outgrowth in *Sort1*^*−/−*^ neurons compared to WT (Figure 6C).

To verify whether PGRN can increase outgrowth in the absence of SORT1 and ultimately whether SORT1 is a neurotrophic receptor for PGRN, we treated primary hippocampal *Sort1*^*−/−*^ and WT neurons with increasing amounts of rPGRN. We found a dose-dependent increase in outgrowth occurs in *Sort1*^*−/−*^ neurons similar to WT (Figure 6D, 6F). Additionally increased branching of these neurons was also seen (Figure 6E, 6G), suggesting PGRN does not use SORT1 to promote outgrowth.

### Missense mutant PGRN alters neuronal morphology

**Figure 7:**
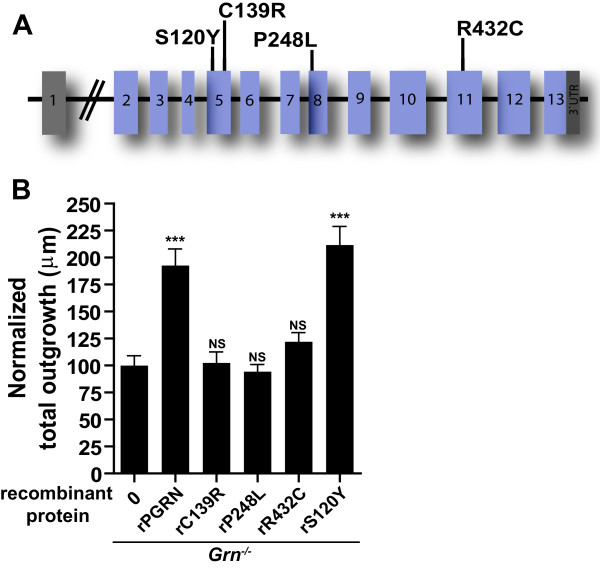
**Specific missense mutations lack the ability to promote outgrowth and branching in primary hippocampal neurons.** (**A**) Schematic representation of the *GRN* gene and location of missense mutations investigated (p.S120Y, p.C139R, p.P248L, p.R432C). (**B**) Bar graph quantification of neurite outgrowth of *Grn*^*−/−*^ hippocampal neurons treated with 125 nM (10 ug/mL) of wild-type rPGRN-His or missense rPGRN-His. Data is presented as the mean ± SEM. **p < 0.01; ***p < 0.001.

While the majority of pathogenic *GRN* mutations lead to the degradation of mutant mRNA and/or cause *GRN* haploinsufficiency, some missense mutations have been reported to disrupt PGRN processing (p.C139R) and secretion (p.R432C, p.P248L), when compared to WT PGRN [7,21]. The p.S120Y *GRN* mutation has been found in controls and believed to be non-pathogenic [22]. To determine whether these missense mutations also disrupt the neurotrophic function of PGRN, we generated recombinant PGRN proteins harboring the aforementioned missense mutations (Figure 7A) and assessed their effect on neurite outgrowth in *Grn*^*−/−*^ neurons. As expected, rPGRN containing p.S120Y, a mutation observed in controls, promoted neurite outgrowth to a similar extent as WT rPGRN (Figure 7B). In contrast, rPGRN with p.C139R, p.R432C and p.P248L mutations failed to stimulate neurite outgrowth (Figure 7B), suggesting these mutations disrupt PGRN function and may indeed be pathogenic and contribute to the disease phenotype observed in patients carrying these mutations.

## Discussion

While loss of PGRN is a major cause of FTLD-TDP, it remains unclear how this loss leads to neurodegeneration. Recent studies suggest that PGRN may function as a protective neurotrophic factor regulating neuronal survival and outgrowth in cortical/motor primary neurons, immortalized cell lines, and zebra fish embryos [10,11,23-25]. The mechanisms underlying these effects have not been fully determined, nor is it known how *GRN* missense mutations and SORT1, a PGRN neuronal receptor, influence PGRN’s neurotrophic properties.

In the current study, we examined the effects of PGRN loss in primary neuronal cultures from *Grn*^*−/−*^ mice. Our data show that neuronal outgrowth and branching in *Grn*^*−/−*^ primary cultures are significantly reduced compared to WT cultures. We also demonstrate that PGRN promotes neurite outgrowth independent of Sort1. Such findings advance our knowledge beyond previous studies, and strongly suggest another receptor(s) modulates PGRN-specific neuronal outgrowth and branching.

Since the discovery of the first *GRN* mutations, several groups have developed various *Grn*^*−/−*^ mouse models [26-28]. While the murine models recapitulate several pathological features of FTLD patients, such as increased gliosis and TDP-43 positive inclusions, no two models are the same, likely due to different backgrounds strains or strategies used to knockout *Grn*. For our experiments, we used primary hippocampal cultures prepared from *Grn*^*−/−*^ mice generated by Kayasuga et al. [27], with the knowledge that these mice display accelerated brain aging due to increased accumulation of lipofuscin, in addition to enhanced gliosis and neurodegeneration when compared to WT mice [29]. These *Grn*^*−/−*^ mice also mimic behavior changes similar to FTLD patients, including increased aggression, decreased social interaction and impaired learning and memory [27,30]. Even though our mice display increased ubiquitin staining, they are not positive for TDP-43, the neuropathological hallmark of FTLD with GRN mutations. Yin et al developed a *Grn*^*−/−*^ mouse displaying amplified inflammation in addition to increased ubiquitin staining positive for TDP-43 [26]. Increased TDP-43 accumulation is also observed in *Grn*^*−/−*^ neurons treated with the proteosome inhibitor MG132, suggesting that these neurons were more vulnerable to cellular stresses than WT [31]. *GRN* haploinsufficiency cell culture models generated similar results, where *GRN* knockdown using siRNA resulted in TDP-43 cleavage; however, downregulation of *GRN* for short time points does not produce TDP-43 cleavage [32,33].

FTLD patients with *GRN* mutations develop severe atrophy of the frontal and temporal lobes of the brain [34]. Therefore, understanding the role of PGRN in neuronal health and function, as well as regulating levels are important for future studies. Recent efforts using *Grn*^*−/−*^ mice have helped shed new light on the many functions of PGRN. Petkau and colleagues determined that *Grn*^*−/−*^ mice have reduced synaptic connectivity and altered LTP, in addition to altered morphology and reduced spine density of apical dendrites of the hippocampus of aged mice [28]. Additional studies in neurons with reduced *GRN* using siRNA revealed decreased neuronal arborization and synaptic density in addition to abnormal transmission at the synapses [31]. These findings suggest that loss of PGRN alters morphology of neurons leading to synaptic changes, which ultimately may cause neurodegeneration.

To further characterize our mice, we compared WT and *Grn*^*−/−*^ cultures and found *Grn*^*−/−*^ primary hippocampal neurons display significantly shorter neurites and fewer branch points compared to WT neurons. Importantly, this phenotype is reversed when neurons are supplemented with PGRN via recombinant or viral methods. While rAAV1-PGRN transduction leads to the intracellular expression of PGRN, ELISA-based quantification of PGRN from media indicated that extracellular PGRN levels increase in a dose-dependent manner following rAAV1-PGRN transduction. Normal physiological ranges for human PGRN are approximately 100–400 ng/mL and 8–12 ng/mL in the PNS and CNS respectively; however, in our WT primary cultures we determined that normal levels are approximately 3.5 ng/mL [32,33]. Increasing doses of rAAV1-PGRN elevated exogenous PGRN levels to 3-15 ng/ml. These studies demonstrate that rAAV1-PGRN transduction resulting in 15 ng/mL PGRN within the media, is sufficient to restore outgrowth and branching of *Grn*^*−/−*^ cultures to WT conditions. We also showed this phenotype can be rescued with the addition of rPGRN and in fact we were able to stimulate outgrowth and branching beyond normal conditions revealing the effective neurotrophic abilities of PGRN. The rPGRN doses exceed the normal range, however, it is difficult to assess what amount of the rPGRN is truly active or degraded as a result of protein purification or normal degradation of the recombinant protein in cell culture models over time.

Our results suggest that PGRN functions as a neurotrophic factor involved in proper neuronal morphology and function. Considering that PGRN is a secreted protein, extracellular PGRN may be responsible for initiating signaling events that promote neurite development. For example, we demonstrate that decreased outgrowth and branching is reversed by the supplementation of PGRN. Given the variety of *Grn*^*−/−*^ models now available, it would be of great interest to determine if model-specific characteristics, such as synaptic abnormalities, altered spines and dendrites, as well as, TDP-43 pathology can be rescued with exogenous PGRN treatment.

In the periphery, PGRN is cleaved into mature granulins by extracellular proteases [15,16], similar to other neurotrophins, such as nerve growth factor (NGF) and brain-derived neurotrophic factor (BDNF), which are synthesized as precursors and proteolytically cleaved to form mature neurotrophins [34]. Our data suggest that rPGRN is being cleaved within primary neuronal culture media (Figure 3A), therefore we hypothesized that cleaved PGRN, or granulins, are responsible for inducing its neurotrophic properties. SLPI, a protease inhibitor, is known to bind PGRN or elastase to prevent PGRN cleavage [15,16]. To determine if full length or cleaved Pgrn is neurotrophic, we treated WT and *Grn*^*−/−*^ primary neurons with and without rSLPI. Our findings show that rSLPI is able to prevent outgrowth in WT cultures; however, this is not seen in knockout cultures suggesting rSLPIs specificity for Pgrn. Van Damme et al. also reported that SLPI abolishes PGRN-enhanced survival and neurite outgrowth in cortical neurons [11]. More importantly, we show that individual granulins C and E have the ability to promote neuronal outgrowth, further emphasizing that PGRN cleavage is required for neurite outgrowth and branching. While we only tested granulins C and E, for future studies it will be important to determine the function of physiologically relevant granulin products, since previous reports suggest that they may have differential effects [16].

Given that previous studies determined the C-terminus tail of PGRN (QLL) binds to the beta-propeller of SORT1 [35], we decided to analyze SORT1 induced uptake of rPGRN. When generating rPGRN, we tagged our peptide using a 6 His tag at either the C-terminus (rPGRN-HIS) or N-terminus (HIS-rPGRN) for purification. His-rPGRN becomes endocytosed by SORT1 overexpression; however, we believed the His tag on rPGRN-His would prevent SORT1 from endocytosing it. Furthermore, we wanted to determine if these two versions of rPGRN have similar neurotrophic properties, since only His-rPGRN interacts with SORT1. Indeed, when we compared His-rPGRN and rPGRN-His in WT cultures, we observed a very similar dose-dependent increase in outgrowth suggesting the neurotrophic properties are not due to the location of the His-tag nor to SORT1.

The impact of individual granulins’ neurotrophic functions and our previously mentioned data suggest that PGRN may be acting independently of SORT1 to promote neurite outgrowth. Our *Sort1*^*−/−*^ cultures exhibit increased levels of PGRN within the media, a finding that is consistent with previous reports demonstrating the ability of SORT1 to regulate PGRN levels [17,35]. These cultures also display a decrease in neuronal outgrowth, which may be puzzling considering the increased Pgrn levels in the media. However, other groups have also reported this phenomenon and point to SORT1’s interaction with tropomyosin-receptor-kinase (Trk) receptors as the cause. Basically, it was determined that SORT1 is involved in the anterograde transport of Trk receptors to nerve endings, where the receptors induce trophic signaling [36]. We believe the decreases in outgrowth in *Sort1*^*−/−*^cultures cultures are caused by the loss of trophic support resulting from Trk receptor misguidance. Furthermore, we observed that by adding increasing amounts of PGRN to *Sort1*^*−/−*^cultures, outgrowth and branching increased in a dose-dependent manner similar to WT neurons. Such findings further suggest PGRN-induced outgrowth occurs independent of SORT1.

Pinpointing exactly which receptor is responsible for PGRN-induced neuronal outgrowth has not yet been determined and will be the aim of future studies. Tang et al, recently discovered PGRN also binds to TNFR [18], and when bound to TNFR, PGRN antagonizes and prevents downstream TNFα-mediated inflammatory signaling. One could speculate that the loss or reduced levels of PGRN long-term will enhance cell death/pro-inflammatory signaling that could be detrimental to neuronal viability. It may be possible that increases in PGRN mitigate the TNFα response to the extent that we observe neurotrophic effects. In such a case, excess PGRN may keep the TNFR signaling in check whereas deficits in PGRN favor a TNFα-mediated response resulting in reduced outgrowth and ultimately neuronal death. Given that PGRN promotes outgrowth independent of SORT1, our data suggest another receptor(s) is involved in PGRN-induced neuronal outgrowth and may be responsible for maintaining neuronal integrity.

Most pathogenic mutations in PGRN result in a loss of functional PGRN caused by nonsense, splice-site and frameshift mutations, as well as by mutations that disrupt proper translation and secretion [4-6]. However, there are several missense mutations of unknown pathogenicity. Lack of functional data currently restricts our understanding regarding the possible contribution of these variants to disease pathogenesis. To further elucidate the role missense mutations with undetermined pathogenicity play in causing a loss of neurotrophic function, rPGRN containing missense mutations were generated and analyzed in hippocampal neuronal cultures. Among the missense mutations studied, two (p.P248L and p.R432C) were reported to lead to decreased secretion, and one (p.C139R) reduces PGRN production, stability and patient plasma levels [7,25,32]. p.S120Y, is unlikely to be pathogenic since it is found in control patients [37,38] was found to promote neurite outgrowth similar to WT rPGRN. In contrast, rPGRN with p.C139R, p.R432C or p.P248L mutations did not stimulate neurite outgrowth, suggesting that these mutations adversely affect PGRN function and may be pathogenic, which would be consistent with previous genetic studies [39,40].

## Conclusions

Taken together, our findings indicate that PGRN is required for the proper development of neurites in primary hippocampal neurons. Our results suggest neurite outgrowth and branching are regulated by extracellular cleaved granulins. We determined that PGRN induces outgrowth independent of SORT1, which indicates that PGRN uses a different receptor to promote neuronal morphology. While pathogenic mutations cause decreases in PGRN levels, certain *GRN* missense mutations abolish the neurotrophic properties of PGRN, providing a putative link between these mutations and development of FTLD-TDP.

## Methods

### Mice

*Grn*^*−/−*^ mice have been previously described [27,29]. *Sort1*^*−/−*^ mice were generated and previously described [41-43]. Timed breeding cages of C57/BL/6, *Grn*^*−/−*^ and *Sort1*^*−/−*^ were set up for primary neuronal cultures. All rodent specimens were obtained from animals handled by procedures approved by the Mayo Clinic Institutional Animal Care and Use Committee.

### Primary neuronal culture

Primary neuronal cultures from hippocampi or cortex were prepared from postnatal day 1 mouse pups and stored at 4°C in HIBERNATE™ A media without calcium (BrainBits), supplemented with B27 (Invitrogen), 0.5 mM GMAX (GIBCO), and gentamicin (GIBCO). Excised hippocampi or cortex were digested in papain (2 mg/mL; Fisher Scientific), triturated with a Pasteur pipet (bore size 0.8–1 mM), centrifuged to collect cell pellet, and resuspended in Neurobasal A (Invitrogen), supplemented with B27, GMAX, gentamicin, and bFGF (Invitrogen). Following determination of cell number, neurons were plated on poly-D-lysine-coated coverslips within 24-well plates for immunocytochemical studies (seeded at a density of 2.5 × 10^4^ cells/coverslip), or seeded on poly-D-lysine coated 6-well plates for immunoblotting at a seeding density of 3 × 10^5^ cells/well. Cultures were allowed to mature for 10 days. Media was changed on the third and seventh days. For experiments using recombinant protein, cells were treated on day 4 and day 7 after media change.

### Recombinant PGRN production

Human progranulin (PGRN) full-length cDNA including the 5’ signal peptide sequence was subcloned into the *Nhe* I and *Age* I restriction sites of the pcDNA4/V5-HisA vector such that a 6 His tag was fused at the carboxyl-terminus of the PGRN sequence. A PGRN stable cell line was generated by transfecting HEK293 cells with the pcDNA4/PGRN-6His DNA and missense PGRN-6His DNA in a 6-well culture plate format. PGRN expression plasmids were sequence verified using ABI3730 with Big Dye chemistry following manufacturers’s protocol (Applied Biosystems, Foster City, CA, USA). Stable transfectants were selected for 4 weeks in complete culture medium supplemented with 400 μg/ml zeocin. Surviving clones were individually picked, expanded and tested for the PGRN expression. The clone that secretes the highest level of PGRN protein into media was used for recombinant PGRN (rPGRN) production. Standard Ni-NTA (Qiagen) affinity purification procedures based on manufacturer’s instruction were applied to purify the rPGRN protein (Lee et al. 2005).

### rAAV1 production

rAAV1-PGRN, rAAV1-SORT1 and rAAV1-GFP was prepared by standard methods. Briefly, AAV vectors expressing PGRN, SORT1 or GFP under the control of the cytomegalovirus enhancer/chicken β-actin promoter, a woodchuck post-transcriptional regulatory element, and the bovine growth hormone, poly(A), were generated by plasmid transfection with helper plasmids in HEK293T cells. All plasmids were sequence verified as described above. Forty-eight hours after transfection, the cells were harvested and lysed in the presence of 0.5% sodium deoxycholate and 50 U/ml Benzonase (Sigma, St. Louis, MO) by freeze thawing, and the virus was isolated using a discontinuous iodixanol gradient. The genomic titer of each virus was determined by quantitative PCR.

### Immunocytochemistry

Primary neurons on coverslips were fixed in methanol, permeablized with 0.5% Triton-X-100 in PBS, washed, blocked in 5% milk-PBS for 1 hour and incubated overnight in anti-MAP2 (1:500, Sigma-Aldrich) or anti-progranulin (1:500, Invitrogen) diluted in 5% milk-PBS. Coverslips were then washed and incubated for 2 hours in goat anti-rabbit Alexa Fluor 568 (1:1000, Molecular Probes) or goat anti-mouse Alexa Fluor 488 (1:1000, Molecular Probes). Coverslips were then incubated for 10 minutes in Hoescht 33258 (1:10000, Invitrogen), washed and mounted onto microscope slides by Fluormount-G (Southern Biotech).

### Image acquisition and quantification

All images were captured using a Zeiss LSM 510 META confocal microscope. Neuronal morphology measurements were determined using MetaMorph version 7.1 (Molecular Devices, Downingtown, PA). In each experiment, 50–100 neurons sampled from randomly selected fields were analyzed and repeated in at least three independent experiments. Statistical analysis was performed using GraphPad Prizm 4 by comparing means of different groups using *Student’s-t* test or ANOVA followed by post hoc Kruskal-Wallis test and Dunn's Multiple Comparison Test. Error bars indicate *p < 0.05; **p < 0.01; ***p < 0.001.

### Western blot and ELISA

Cells from 6 well plates were lysed in lysis buffer consisting of Co-IP buffer (50 mM Tris–HCl, pH 7.4, 1 M NaCl, 1% Triton-X-100, 5 mM EDTA) plus 1% SDS, PMSF, and both a protease and phosphatase inhibitor mixture. The protein concentration of cell lysates was measured using a BCA assay (Pierce). Samples were prepared in Laemmli’s buffer, heated for 5 min at 95°C, and equal amounts of protein were loaded into 10-well 10% Tris-glycine gels (Novex). After transfer, blots were blocked with 5% nonfat dry milk in TBST (TBS plus 0.1% TritonX-100) for 1 h, and then blots were incubated with anti-mouse progranulin (1:1000, R&D systems), anti-human progranulin (1:1000, Invitrogen), mouse monoclonal GAPDH antibody (1:10000, Biodesign) overnight at 4°C. Membranes were washed three times for 10 min in TBST and then incubated with donkey anti-rabbit, anti-mouse or anti-sheep IgG conjugated to horseradish peroxidase (1:2500; Jackson ImmunoResearch) for 1 hour. Membranes were washed three times each for 10 min, and protein expression was visualized by ECL treatment and exposure to film. Human PGRN ELISA (R&D Systems) and Mouse Pgrn ELISA (Enzo Life Sciences) was used according to manufacture protocol on media collected from rAAV1-PGRN treated cultures.

### Enzymatic digestion of rPGRN by elastase

30 μg of wild-type recombinant PGRN or mutants PGRN protein incubated on ice was mixed with reaction buffer (100 mM Tris–HCl, 300 mM NaCl, pH 7.5) and 0.05 U/ml purified elastase (Athens Research and Technology) in a total reaction volume of 100 μl. The enzyme reaction was initiated by incubation at 37°C. 10 μl of the reaction mixture was sampled every 5 minutes and the reaction was immediately quenched by adding equal amount of 2 × SDS sample buffer. The reaction was allowed to proceed until 35 minutes after time zero. A separate reaction with the addition of recombinant SLPI at 13.3 μM was also prepared and preceded for 35 minutes at 37°C. The samples were analyzed by SDS-PAGE, post-stain with sypro ruby solution (Sigma) and imaged by a gel doc station equipped with a UV-transilluminator.

### List of abbreviations

PGRN: human progranulin; Pgrn: mouse progranulin; *GRN*: human progranulin gene name; *Grn*: mouse progranulin gene name; *Sort1*: mouse sortilin gene; SORT1: human sortilin 1; Sort1: mouse sortilin 1; FTLD: Frontotemporal lobar degeneration; TDP-43: transactive response DNA-binding protein 43; AD: Alzheimer’s disease; AAV: adeno-associated virus.

## Competing interests

The authors declare that they have no competing interests.

## Authors’ contributions

JG carried out the primary neuronal cell culture, immunohistochemistry, Image acquisition and quantification, designed the study and wrote the manuscript. CLee generated recombinant proteins and performed elastase digest. CC provided intellectual input for primary neuronal cell culture and design of study. NF and RR preformed ELISA experiments. CS edited the manuscript. CLink provided intellectual for design of study. JL and AN provided intellectual input for mice. LP is the principle investigator and designed study. All authors read and approved the final manuscript.

## Supplementary Material

Additional file 1 **Figure S1.** rSLPI prevents rPGRN induced outgrowth and branching. **A-B**. Bar graph quantification of normalized total outgrowth (**A**) and branching (**B**) in primary hippocampal cultures treated with rPGRN ± rSLPI. Data is presented as the mean ± SEM. Click here for file
